# Next-Generation Vaccines: Nanovaccines in the Fight against SARS-CoV-2 Virus and beyond SARS-CoV-2

**DOI:** 10.1155/2023/4588659

**Published:** 2023-05-04

**Authors:** Maluta Steven Mufamadi, Mpho Phehello Ngoepe, Ofentse Nobela, Nhlanhla Maluleke, Bafedile Phorah, Banele Methula, Thapelo Maseko, Dipuo Ingrid Masebe, Hazel Tumelo Mufhandu, Lebogang Maureen Katata-Seru

**Affiliations:** ^1^DSI-Mandela Nanomedicine Platform, Nelson Mandela University, Gqeberha 6059, South Africa; ^2^Nabio Consulting (Pty) Ltd., Pretoria 0183, South Africa; ^3^Department of Microbiology, North-West University, Mafikeng 2745, South Africa; ^4^Department of Chemistry, North-West University, Mafikeng 2745, South Africa

## Abstract

The virus responsible for the coronavirus viral pandemic is the severe acute respiratory syndrome coronavirus 2 (SARS-CoV-2). Emerging SARS-CoV-2 variants caused by distinctive mutations within the viral spike glycoprotein of SARS-CoV-2 are considered the cause for the rapid spread of the disease and make it challenging to treat SARS-CoV-2. The manufacturing of appropriate efficient vaccines and therapeutics is the only option to combat this pandemic. Nanomedicine has enabled the delivery of nucleic acids and protein-based vaccines to antigen-presenting cells to produce protective immunity against the coronavirus. Nucleic acid-based vaccines, particularly mRNA nanotechnology vaccines, are the best prevention option against the SARS-CoV-2 pandemic worldwide, and they are effective against the novel coronavirus and its multiple variants. This review will report on progress made thus far with SARS-CoV-2 vaccines and beyond employing nanotechnology-based nucleic acid vaccine approaches.

## 1. Introduction

In December 2019, a deadly virus emerged in Wuhan City in China, and by 11 March 2020, the infectious virus had spread rapidly, and the World Health Organization (WHO) declared it a pandemic. The pandemic infection was termed COVID-19, and it was discovered to be caused by a fatal coronavirus, which the WHO eventually categorized as severe acute respiratory syndrome coronaviruses 2 (SARS-CoV-2) [1]. The viral threat has as a result that caused tremendous health and economic losses. As of 19 December 2022, the current SARS-CoV-2 cases reported are about 657,840,721 confirmed cases, including 6,672,072 deaths worldwide. Coronaviruses are enveloped RNA viruses that belong to the *Coronaries* family [2]. They are named for the crown-like appearance of the surface glycoproteins when viewed under an electron microscope and are known to infect both humans and some animals [3]. The clinical symptoms of coronaviruses range from mild illnesses much like cold or flu to more severe symptoms like the Middle East respiratory syndrome (MERS) and severe acute respiratory syndrome (SARS) which leads to acute respiratory distress syndrome (ARDS) [4]. The spike (S) glycoprotein, small envelope (E), matrix (M), and nucleocapsid (N) proteins, among others, are involved in the pathogenesis of SARS-CoV-2 [5]. Since it facilitates viral entry into the host cell during the infection phase, the S protein is the best target for vaccine development. Scientists worldwide are working tirelessly to develop effective vaccines and other treatment options for SARS-CoV-2.

In the fight against the SARS-CoV-2 pandemic, nanotechnology-based applications can be used in a variety of ways, including the invention of a sensitive, rapid, and precise diagnostic tool for SARS-CoV-2, the use of nanomaterials to deliver antiviral agents, and the creation of efficient environmental disinfectants [6]. Nanomedicine is a medical application of nanotechnology used to diagnose, prevent, and treat diseases and to better understand the intricate mechanisms of these diseases [7]. Nanomedicine has been at the forefront of developing SARS-CoV-2 vaccines and therapy; so far, several nanotechnology-based SARS-CoV-2 vaccines have been approved and administered around the world as the first line defence against SARS-CoV-2 [8]. Adjuvants and antigens are delivered via novel platforms in nanomedicine, which alter innate and adaptive immunity to trigger potent antiviral responses.

Nanotechnology-based vaccines (nanovaccines) are an emerging new class of vaccines that consists of nanoparticles that directly target the site of infection in the body. Nanocarriers such as lipids, polymeric nanoparticles, and liposomes have emerged as an effective vaccine vehicle against infectious diseases [9]. For three primary reasons, nanoparticles may be included in vaccine formulations: increased immunogenicity as immunomodulatory carriers to elicit an immune response, increased antigen stability by preventing premature degradation by proteases and nucleases, and targeted delivery using nanoparticles as delivery systems to stimulate antigen uptake and processing by antigen-presenting cells [10]. Additionally, by inducing both humoral immunity (antibody-mediated immunity) and cell-mediated immune responses, nanovaccines can enhance vaccine effectiveness [11]. Guerrini et al. [12] have described methods for the characterisation of nanoparticle-based vaccines, including physical, chemical, stability, in vitro immunogenicity, in vitro toxicity, and in vivo preclinical testing assays.

**Figure 1:**
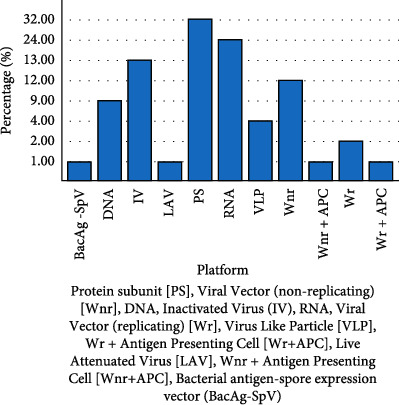
Current progress in vaccine development.

Based on the idea that when a virus infects a cell, the antigen encoded by the nucleic acid is produced, resulting in a cell-mediated response; nucleic acid vaccination can be employed to protect against infections [13]. Nanocarriers are needed because naked RNA is easily degraded and needs to be protected during delivery. The DNA and messenger RNA (mRNA) vaccines are examples of nucleic acid-based vaccines [14]. The SARS-CoV-2 S protein is produced inertly by cells under the control of genetically modified mRNA in the mRNA vaccines. After vaccination, the immune response cells produce S protein fragments and present them on cell surfaces. DNA vaccines use a plasmid containing an antigen-coding DNA sequence to generate immune responses. In this case, SARS-CoV-2, a bacterial plasmid, uses the DNA sequence encoding the spike protein of SARS-CoV-2 to produce the neutralizing antibody protection against coronavirus infections. This method is known to be effective and stimulate both humoral and cell-mediated immune responses [15]. Several types of SARS-CoV-2 vaccines are currently in use, and others are still in the preclinical and clinical stages (Figure 1). Included in this are inactivated vaccines (noninfectious) and live-attenuated vaccines (weakened virus), as well as nucleic acid vaccines (mRNA and DNA), adenovirus-based vector vaccines (carrier virus), recombinant subunit vaccines (viral proteins), and virus-like particles (VLPs) (viral capsid) [16].

Inactivated viruses are commonly used methods for vaccine development (e.g., influenza and poliovirus). Inactivated (formaldehyde, glutaraldehyde, ultraviolet, or gamma rays) SARS-CoV-2 strains from hospitalized patients were observed to lead to controllable viral infection in animals at a low dose, while at high dosing led to undetectable viral loads [17]. The deletion of the accessory proteins from SARS-CoV-2 strains from hospitalized patients can be used to attenuate SARS-CoV-2, which is known to have low production costs and produce long immune durability [18]. The drawback of inactivated vaccines is that during the inactivation process, the virus antigens and epitopes may be destroyed, resulting in a reduced immune response [19].

Adenoviruses can be used as viral vectors in vaccine development employing deletion of viral propagation genome and then inserting into the adenovirus genome a SARS-CoV-2 gene encoding the targeted epitopes. Although adenovirus-based vector vaccines are easy to purify to high titter and also inexpensive, they are associated with adverse effects due to preexisting antiadenovirus immunity [20]. Virus-like particle (VLP) technology uses viral capsid proteins or replication-defective virus particles, which are like nanoparticle vaccines because they can also be used for drug delivery [21]. Nanotechnology-based vaccines utilizing nucleic acid and protein subunit vaccines are the focus in this review.

### 1.1. Nanotechnology-Based Vaccines for SARS-CoV-2

The use of vaccines has shown great impact over the years as a prominent preventive measure against infectious diseases [22]. The vaccination process involves the stimulation of the immune system aided by antigenic materials. Over time, the required immune response could not be achieved due to poor delivery of the antigen to the desired site. This has led to many studies around using an antigen carrier [23]. Nanotechnology has revolutionized the manufacture of vaccines, but it has been challenging to identify a suitable carrier. Nanotechnology has come with the promise of increasing vaccine efficacy and reducing the chances of lethal side effects. The use of the nanomaterials as a delivery vehicle provides them with the added advantage over traditional vaccines (e.g., inactivated and live-attenuated strains) that still require cultures and are expensive. Many types of nanoparticles are being exploited as nonviral vectors and/or as adjuvants [24]. Some of the nanoparticles used are polymeric nanoparticles, inorganic nanoparticles, virus-like nanoparticles, liposomes, and self-assembled protein nanoparticles [25]. Nanoparticles can be fabricated from synthetic or natural materials. There is an ongoing debate about which nanoparticles are most effective as a vaccine vehicle [26]. The synthetic nanoparticles allow for modifications of the physicochemical properties and incorporation of a wide range of molecules, including antigens, hence increasing their effectiveness in targeted delivery [27]. Nanoparticles from natural polymers are more stable and have higher biocompatibility in cells.

Nanoparticles have many benefits, including efficient vaccine delivery, vaccine delivery to specific cells, such as immune response cells, ease of movement through tissue barriers, increased bioavailability, and effective delivery of antigen and adjuvant to antigen-presenting cells (APC) and natural killer (NK) cells, despite the possibility that their composition could affect their efficacy. Due to their small size, nanoparticles have a fairly high diffusion capacity, allowing for ease of administration [28]. As a result, nanovaccines can be administered subcutaneously, intravenously, intranodal, and intramuscularly. Oral and intranasal administration of the nanoparticles to mucosal sites is also suggested and investigated as nanoparticles have been shown to have the capacity to penetrate capillaries and mucosal surfaces [29].

Nanovaccines have gained much attention due to the recent SARS-CoV-2 disease outbreak [30]. Nanovaccines have shown to be the preferred nonviral vector as compared to viral vectors, such as the rVSV-ZEBOV vaccine against Ebola [31]. Nanotechnology-based mRNA vaccines are among the licensed SARS-CoV-2 vaccines and are authorised for emergency use worldwide [32]. Khurana et al.'s [26] study suggested that the success of nanotechnology-based vaccines might play a critical role in the success of the delivery of nucleic acid-based therapeutics. The advancement of nanotechnology-based mRNA vaccines for COVID-19 opens an opportunity for the groundbreaking development of vaccines against other complex diseases such as HIV/AIDS, influenza, Zika, malaria, and Ebola [33].

### 1.2. DNA Nanotechnology-Based Vaccine for SARS-CoV-2

DNA vaccines, also known as plasmid DNA, utilise a DNA molecule carrying genetic information to instruct the cells to produce the antigen [34]. An antigen is a molecule, normally a protein that is present outside a foreign particle that can stimulate an immune response. Nanoparticles facilitate the transport of this vaccine to the cells as carriers or adjuvants [35]. The nanoparticles allow the plasmid DNA vaccine to easily enter the cells and offer protection against degradation [5]. Nanoparticles made from lipid materials are compatible with our bodies. The lipid nanoparticles are made up of a lipid material that is like the one in the cell membranes, which makes it easier to facilitate DNA entry into targeted cells. The basic structure of the DNA vaccine may be divided into two distinct units. A transcription complex unit is composed of a promoter sequence that induces expression in a variety of cells, gene coding for the target antigen, and polyadenylation sequences to enhance stability [36].

The production unit comprises the replication origin and microbial sequences for selection and plasmid amplification [37]. Plasmids are smaller circular pieces of DNA that replicate independently. *Escherichia coli* is the most common microorganism utilised for the production of plasmid DNA [38]. The gene of interest is first synthesised and incorporated into the plasmid through genetic engineering. The technique of DNA synthesis eliminates the expensive isolation and purification of the protein from the infectious antigen. Subsequently, the plasmid DNA is incorporated into a bacteria or yeast replication. The engineered DNA is then extracted, purified, and prepared for administration [39].

**Figure 2:**
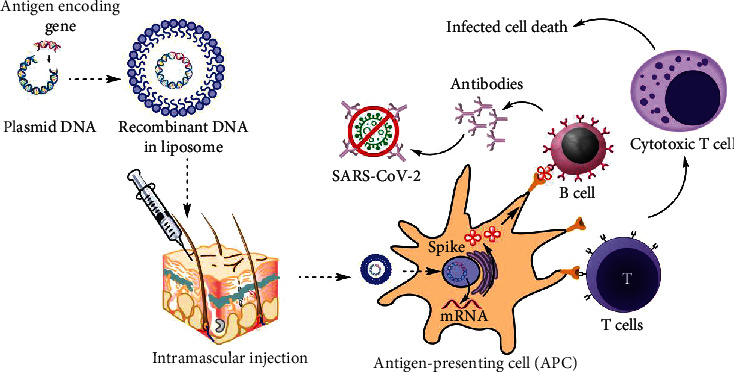
DNA vaccine mechanism of action.

The DNA strand must go through the nucleus to be transcribed into the mRNA (Figure 2). However, it does not interfere with the DNA of the host by carrying genetic information specifically for the antigen. Subsequently, the immune system will recognize it as a foreign particle and produce neutralizing antibodies that bind to a specific antigen. After neutralization, the immune system would have learnt the antigen and memory cells will store memory of this antigen [40]. In the future, if the same foreign particle invades the body, the immune system will recognize it and attack aggressively before the foreign particle can spread and cause disease. DNA vaccines can induce both humoral and cellular immunity, which is based on the production of antibodies and the production of macrophages and cytokines, respectively.

DNA vaccination has been extensively applied in veterinary medicine for the prevention of infections [41]. The evaluation of DNA vaccines to prevent various human infections such as influenza, cancer, and HIV-1 started a long time ago [42]. Although the results of the early studies were inadequate, there are successful DNA vaccines, including those currently in clinical trials. There are recent developments in the application of DNA vaccines for the prevention of infections, including the pandemic of our time and SARS-CoV-2 (Table 1). ZyCoV-D (Zydus Lifesciences, Ahmedabad, India) is approved for clinical use in India. Undesirable viral mutations may occur naturally, resulting in different variants. Emerging variants may be easily transmissible or resistant to vaccines; however, the nanovaccines may still be effective. The SARS-CoV-2 variants B.1.1.7 (UK) and B.1.351 (South Africa) which have an extensive mutation on the spike protein were observed to be resistant to monoclonal antibodies of SARS-CoV-2 vaccinated individuals [43]. A DNA vaccine that targets the spike glycoprotein of SARS-CoV-2 has been proven in preclinical research to generate humoral and cellular immune responses, indicating that it is an effective and safe strategy for fighting the SARS-CoV-2 pandemic [44].

### 1.3. Messenger RNA (mRNA) Vaccines for SARS-CoV-2

Approximately three decades ago, scientists began exploring the possibilities of making vaccines simpler than the conventional techniques of using weakened or inactive fragments and purified or genetically engineered viral proteins of an antigen to trigger an immune response [46]. The worth of the time spent on this research has led to the pioneered emergency use authorisation (EUA) of a new generation of vaccines used to control the current SARS-CoV-2 pandemic. To mobilize innate and adaptive immune responses against SARS-CoV-2, lipid nanoparticles were used [47]. Lipid nanoparticles are of interest as they pose no potential risk of genome integration; they do not require entry into the nucleus and can be developed very rapidly and easily compared to traditional vaccines. The novel vaccines are made up of mRNA strands packaged neutrally charged and lipid-based nanoparticle delivery systems [48] and are first in line for vaccines to have ever been developed, from preclinical to the clinical phase, at a fast rate as observed recently.

The current mRNA vaccines target the same antigen that incorporates mRNA encoding the full-length transmembrane anchored S protein. The genetic sequence is slightly altered with the introduction of two proline (2P) substitutions to stabilise the prefusion conformation of the glycoprotein [49]. The mRNA vaccination approach's most notable characteristic is that the proteins are created by host cells, much like in a situation of viral infection that occurs naturally. The S glycoprotein is effectively exposed to B cells in its antigenically natural prefusion conformation as a result of the produced proteins going through the same posttransactional processing and becoming engulfed as a trimer in the membrane of mRNA-transfected cells [50].

**Figure 3:**
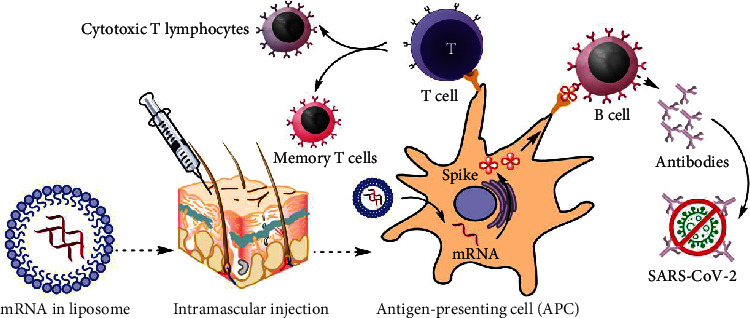
mRNA vaccine mechanism of action.

A vaccination needs an adjuvant and a pathogen-specific immunogen to induce adaptive immunity [51]. The latter triggers the innate immune system and offers the second signal for T-cell activation. An ideal adjuvant promotes innate immunity without causing chronic inflammation that could have negative side effects [52]. Because RNA has inherent immunostimulatory properties, the mRNA used in mRNA vaccines can serve as an immunogen by encoding an antigen of interest and as an adjuvant due to intrinsic immunostimulatory properties of RNA [53]. An important component of the innate immune response to viruses is the recognition of single-stranded RNA (ssRNA) and double-stranded RNA (dsRNA) upon entry into cells by several endosomal and cytosolic innate sensors (Figure 3). TLR3 and TLR7, endosomal Toll-like receptors, bind to ssRNA in the endosome, whereas MDA5, RIG-1, NOD2, and PKR, constituents of the inflammasome, bind to ssRNA and dsRNA in the cytosol. This causes cellular activation and the generation of type I interferon and several inflammatory mediators [54].

### 1.4. Effectiveness of mRNA Vaccines on the SARS-CoV-2 Alpha and Delta Variants

The world has witnessed a swift growth of SARS-CoV-2 mutations and emerging variants of concern (VOC) and variants of interest (VOI) [55]. The Alpha (B.1.1.7) and Delta (B.1.617.2) SARS-CoV-2 variants have been dominating with alarming transmissible rates, more aggressive symptoms, and carrying a much higher risk of hospital admissions. A study revealed that people who have received two doses of the authorised mRNA vaccines developed antibody responses against the original SARS-CoV-2. The immune response was strong against the Alpha variants but decreased efficacy against the Beta variants. Currently, GEMCOVAC-19 (Gennova Biopharmaceuticals Ltd., Maharashtra, India) is approved in a country like India, TAK-919 (Takeda, Tokyo, Japan) is approved in Japan, Spikevax/mRNA-1273/elasomeran (Moderna, Inc., Massachusetts, USA) is approved in eighty-seven countries, and Comirnaty/tozinameran/BNT162b2 (Pfizer/BioNTech, New York, USA) is approved in 146 countries. A list of nanotechnology-based mRNA vaccines for SARS-CoV-2 under clinical trials and clinical use is illustrated in Table 2.

Any new vaccine design must include preclinical research to evaluate the vaccine's efficacy on the target populations. Animal models that closely mimic human disease can be used to establish immune protection, but they do not always transfer into practical immunological correlates of protection. Immunization-induced amnestic responses can rapidly produce targeted antibodies that can prevent the spread of initial infection by neutralizing the pathogen. Due to the amnestic response requiring booster shots, it has been demonstrated that antibody levels measure immunogenicity rather than necessarily efficacy when measuring the effectiveness of vaccination against HBV surface antigen [56]. This has been noticed with the multiple booster SARS-CoV-2 vaccinations. Recent preclinical studies in the development of vaccines against SARS-CoV-2 are illustrated in Table 3. A study on self-amplifying mRNA SARS-CoV-2 vaccine candidate delivered by lipid nanoparticles (LNPs) in rodent models (mice, rats, and hamsters) was to neutralize the B.1.1.7 (Alpha), B.1.351 (Beta), and B.1.617.2 (Delta) variants [57].

### 1.5. Recombinant Spike Protein Nanotechnology-Based Vaccines for SARS-CoV-2

The spike protein that is found on the viral membrane of SARS-CoV-2 is one of the most important structures of the virus as it plays a vital role when the virus enters a host. Angiotensin-converting enzyme 2 (ACE2) is recognized by the spike protein of SARS-CoV-2 as the cell entry receptor [59]. Scientists have used the spike proteins to create a SARS-CoV-2 vaccine by analysing the structural data and biological function of the SARS-CoV-2 spike protein and its essential receptor-binding domain (RBD) [60]. High-resolution interface mutation scanning and cryo-EM have both been used to confirm the structure of the spike and RBD proteins. To create the vaccine, scientists started by modifying a spike protein gene and inserted it into a different type of virus known as baculovirus and allowed it to infect mucosal cells [61]. As seen on the coronavirus surface, spike proteins formed by infected cells spontaneously joined together to form spikes. Licensed vaccines for diseases like influenza and HPV have already been developed using a similar method of growing and harvesting virus proteins [62].

The recombinant protein nanotech SARS-CoV-2 vaccine candidates use a full length of the wild-type SARS-CoV-2 spike glycoprotein (Table 4) [63]. Novavax Inc.'s NVX-CoV2373 vaccine is an example of recombinant spike protein composed of trimeric full-length SARS-CoV-2 spike glycoproteins and Matrix-M1 adjuvant [64]. NVX-CoV2373 (Novavax) was demonstrated to have 89% efficacy in preventing clinical SARS-CoV-2, despite early clinical studies reporting the vaccine to be successful. However, concerning the B.1.1.7 variant, the efficacy was lowered by a ratio of 1.8 [65]. In contrast, the NVX-CoV2373 vaccine for the B.1.351 variant demonstrated an efficacy of 49% in the prevention of mild, moderate, and severe SARS-CoV-2 [66].

### 1.6. Advantages and Disadvantages of Nanotechnology-Based Vaccines

There are currently two types of nanovaccines: protective (prophylactic) and therapeutic nanovaccines [67]. Protective (prophylactic) nanovaccines are used to prevent different diseases, and several have been approved for trials in humans or undergoing clinical or preclinical trials. Therapeutic nanovaccines have been implicated in the treatments of multiple diseases such as cancer, Alzheimer's disease, hypertension, and inflammatory diseases [68]. Nanovaccines have multiple benefits when compared to traditional vaccines. Unlike traditional vaccines, nanovaccines do not act on the whole body; they can actively target affected tissue and reach lymph nodes easily without using peripheral dendritic cells [69]. The use of nanoparticles as adjuvants for vaccines includes benefits such as stability in blood flow, enhanced immune response, and temperature stability. In addition, they can aid in solubilizing both hydrophilic and hydrophobic compounds and protect antigens against degradation [70].

Nanoparticles can present antigens to effector cells such as macrophages and dendritic cells and modulate the uptake of antigens. Furthermore, they are highly degradable and easy to synthesise and manufacture, as the mature mRNA manufacturing process and formulation platform can produce various vaccines within very short periods [71]. Nanovaccines have fewer risk factors when compared to traditional vaccines. Despite the highlighted benefits, nanovaccines have limitations in vaccine manufacturing due to their toxicity and challenges in presenting native antigens. Additionally, they can build up in cells due to immunizations, which can lead to problems in cases of prolonged exposure [72]. The biodegradable polymer has limited use in solid particle vaccines due to protein entrapment and loss of immunogenicity, while liposomal delivery technologies have a downside in nanovaccine due to aggregation during storage [73].

The mRNA nanotech SARS-CoV-2 vaccine candidates use mRNA encoding the full-length spike protein of SARS-CoV-2 to elicit protective immunity against coronaviruses. Nanotech DNA candidates for the SARS-CoV-2 vaccine make use of a small fragment of bacterial DNA plasmid that encodes the virus' spike protein [74] and nanotech utilizing recombinant proteins; the full-length wild-type SARS-CoV-2 spike glycoprotein is used in SARS-CoV-2 vaccine candidates [75]. Traditional immunization procedures include subcutaneous, intradermal, and intramuscular injections, while oral, nasal, and aerosol vaccination is an alternate technique. Since SARS-CoV-2 readily attaches to the nasal mucosa when inhaled, nasal mucosal immunization is the best approach to SARS-CoV-2 vaccination [76]. Systemic and mucosal immunity can be produced by the SARS-CoV-2 nanovaccines by stimulating the nasal mucosa. Both serum and mucosal antibody immunological responses were seen to be stimulated by a single intranasal administration of the SARS-CoV-2 spike protein (AdCOVID) vaccine [77].

## 2. Post-SARS-CoV-2 Epidemic and Future Perspectives of Nanovaccines

### 2.1. HIV mRNA Vaccine

According to data from The Joint United Nations Programme on HIV/AIDS (UNAIDS), there were approximately 38.4 million HIV-positive individuals worldwide in 2021 [78]. Currently, their mode of treatment for individuals exposed to HIV is through oral preexposure prophylaxis (PrEP) and prolonged combined antiretroviral therapy (cART). Developing an effective HIV vaccine (e.g., VaxSyn, HIVAC-1e, and vCP125) through conventional (recombinant gene expression) means has been difficult due to the high rate of mutation and recombination during HIV viral replication affecting the antibody target viral envelope (Env) glycoprotein [79]. It has been generally lauded for the RV144 vaccine's great effectiveness in preventing HIV-1 acquisition, which peaked at 60% at month 12 but fell to 31.2% by month 42 [80]. Various mRNA HIV vaccine delivery techniques can be used such as electroporation (direct patient application not foreseeable), cationic micelles, cationic nanoemulsion (CNE), cationic lipid nanoparticles, poly (lactic acid) (PLA) nanoparticles, and *ex vivo* loading of dendritic cell (labour intensive and expensive) [81]. Clinical trials have been conducted on dendritic cells loaded with mRNA (e.g., NCT00833781, NCT00381212, and NCT00672191) and bare viral antigenic mRNA (NCT02413645). A phase 1 study (NCT05414786) is currently recruiting participants in South Africa and Rwanda to evaluate the safety and immunogenicity of the eOD-GT8 60mer mRNA vaccine (mRNA-1644). Currently, on the clinical trial website, there are 7 HIV clinical trials for the HIV vaccine based on mRNA technology. Among numerous studies, nanoformulations (e.g., liposomes, lipid nanoparticles, and polymer-based nanoparticles) are widely utilised in the delivery of HIV mRNA vaccine delivery [82].

### 2.2. Cancer Immunotherapy

The mRNA cancer vaccine has been proposed to be able to take the place of other traditional vaccination platforms for cancer immunotherapy due to its high potency, safe administration, quick development potential, and affordable production [83]. The tumour antigens encoding mRNA vaccines can stimulate tumour-specific immune responses by firstly targeting antigen-presenting cells (APC) such as dendritic cells (DCs) to express antitumour antigens which can be recognized by the CD8^+^ T-cells [84]. Nanomaterials play a crucial role in cancer vaccine development as they can act as delivery systems of tumour-associated antigens (TAAs), tumour-specific antigens (TSAs), or peptides (neoantigens) and for targeting specific immune cell types [85]. The nanomaterials can also serve as adjuvants that can directly enhance cellular and humoral immune responses in cancer immunotherapy. Since the late 1990s, the mRNA-based approach of expressing TAAs on DCs has opened the path to the current cell (APCs) and site (e.g., lymph nodes) specific nanovaccine applications for cancer immunotherapy [86]. The A liposomal mRNA vaccine, FixVac BNT111 (BioNTech SE, Mainz, Germany) which can encode 4 different TSAs, was observed to cause antigen-specific cytotoxic T-cell responses among melanoma patients during clinical trials (NCT02410733) [87]. The distinctive feature of mRNA vaccines is that they can be customized based on antigen genetic mutation features for efficient cancer treatment [88]. There are currently various clinical trials for melanoma stage III/IV (BNT111 and mRNA-4157), prostate cancer (BNT112 and CV9103), ovarian cancer (W_ova1 and mRNA-2416), colorectal cancer stage II/III (BNT122), non-small-cell lung cancer (BNT116), and solid tumours (mRNA-4157 and BNT151) based on mRNA-based vaccines [89]. To improve the efficacy of the cancer vaccines, immune checkpoint inhibitors (ICIs) can also be used against nonimmunogenic (diminished cytotoxic killing activity against tumour cells) tumours such as prostate and pancreatic cancers [90].

## 3. Conclusions

In the immediate aftermath of the SARS-CoV-2 pandemic, nanovaccines may be effective in combatting many other infectious diseases, such as the Middle East respiratory syndrome (MERS-CoV), HIV, malaria, and Zika virus, which still require effective and affordable vaccines. In the future, we are more likely to see novel nanotechnologies being used to reformulate old vaccines since nanocarrier-based delivery systems can get the vaccine to target areas, unlike conventional vaccines. The future epidemic is more likely to be confronted with new nanotechnologies due to advances in the field of nanotechnology. Nanovaccines have thus far presented themselves as effective and best to use to tackle this pandemic since there is an urgent need to develop effective and safe vaccines and therapeutics against the rising virus variants. They have demonstrated high efficacy and minimal life-threatening side effects. The greatest prophylactic approach against the SARS-CoV-2 pandemic has been nucleic acid-based vaccines, notably mRNA nanotech vaccines, which are effective against both novel coronavirus and its numerous variants. These nucleic acid-based vaccines are currently being administered worldwide in the fight against SARS-CoV-2. Nanovaccines have presented more advantages compared to traditional vaccines in the fight against SARS-CoV-2.

## Figures and Tables

**Table 1:** DNA nanotechnology-based vaccines: clinical phases update this table (adapted from VIPER Group COVID-19 Vaccine Tracker Team [45]).

Vaccine	Company	Phase	Status
GX-19N	Genexine, Inc.	2/3	Withdrawn
GLS-5310	GeneOne Life Science, Inc.	1/2	Active
AG0302	AnGes, Inc.	2/3	Completed
ZyCoV-D	Zydus Cadila	—	Approved
INO-4800	Inovio	2/3	Terminated
AG0301	AnGes, Inc.	1/2	Completed
COVID-eVax	Takis	1/2	Terminated
Covigenix VAX-001	Entos Pharmaceuticals Inc.	1/2	Recruiting
VB10.2129/VB10.2210	Nykode Therapeutics	1/2	Active
COVIGEN	University of Sydney	1	Active
COVIDITY	Scancell	1	Active
bacTRL-Spike	Symvivo	1	Completed

**Table 2:** Nanotechnology-based mRNA vaccines for SARS-CoV-2 in the clinical phase in some countries and undergoing preclinical trials in others (adapted from VIPER Group COVID-19 Vaccine Tracker Team [45]).

Vaccine	Company	Phase	Status
Gemcovac	Gennova Biopharmaceuticals	—	Approved
Spikevax	Moderna	—	Approved
Comirnaty	Pfizer/BioNTech	—	Approved
TAK-919	Takeda	—	Approved
CVnCoV	CureVac	3	Completed
AWcorna	Walvax	3	Recruiting
ARCT-154	Arcturus Therapeutics Inc.	2/3	Active
mRNA-1273.211	Moderna	2/3	Active
19 mRNA vaccine	CanSino Biologics Inc.	3	Pending
DS-5670a	Daiichi Sankyo Co., Ltd.	1/2	Completed
LVRNA009	AIM Vaccine	3	Pending
BNT162b1	Pfizer/BioNTech	2/3	Completed
BNT162b2s01	Pfizer/BioNTech	2/3	Completed
EXG-5003	Elixirgen Therapeutics, Inc.	1/2	Completed
EG-COVID-003	EyeGene Inc.	1/2	Recruiting
LUNAR-COV19/ARCT-021	Arcturus Therapeutics Inc.	2	Terminated
MRT5500	Sanofi Pasteur	1	Terminated
ChulaCov19	Chulalongkorn University	1/2	Pending
CoV2 SAM (LNP)	GlaxoSmithKline	1	Completed
VLPCOV-01	VLP Therapeutics Japan	1	Pending
LNP-nCOV saRNA-02	MRC/UVRI and LSHTM Uganda Research Unit	1	Recruiting
GRT-R914 (samRNA)	Gritstone bio, Inc.	1	Active
HDT-301	HDT Bio	1	Active
mRNA-1273.351	Moderna	1	Complete
MIPSCo-mRNA-RBD-1	University of Melbourne	1	Recruiting
ABO-CoV.617.2	Suzhou Abogen Biosciences	1	Active

**Table 3:** Nanotechnology-based mRNA vaccines for SARS-CoV-2 in the preclinical phase (adapted from Tian et al. [58]).

Type of candidate vaccine	Company
saRNA formulated in an NLC	Infectious Disease Research Institute/Amyris, Inc.
LNP-encapsulated mRNA encoding S	Max-Planck-Institute of Colloids and Interfaces
Self-amplifying RNA	Gennova
mRNA	Selcuk University
LNP-mRNA	Translate Bio/Sanofi Pasteur
LNP-mRNA	CanSino Biologics/Precision NanoSystems
LNP-encapsulated mRNA cocktail encoding VLP	Fudan University/Shanghai Jiao Tong University/RNACure Biopharma
LNP-encapsulated mRNA encoding RBD	Fudan University/Shanghai Jiao Tong University/RNACure Biopharma
LNP-encapsulated mRNA	University of Tokyo/Daiichi Sankyo
Liposome-encapsulated mRNA	BIOCAD
Several mRNA candidates	RNAimmune, Inc.
mRNA	FBRI SRC VB VECTOR, Rospotrebnadzor, Koltsovo
mRNA	China CDC/Tongji University/Stermina
mRNA in an intranasal delivery system	eTheRNA
mRNA	Greenlight Biosciences
mRNA	IDIBAPS-Hospital Clinic, Spain
mRNA	Providence Therapeutics
mRNA	Cell Tech Pharmed
mRNA	ReNAP Co.
D614G variant LNP-encapsulated mRNA	Globe Biotech Ltd.
Encapsulated mRNA	CEA
Recombinant, prefusion stabilised SARS-CoV-2 spike antigen	Medigen Vaccine Biologics Corp (MVC)/Vaxess Technologies (MIMIX)
ZIP1642 is a self-amplifying RNA vaccine encapsulated in an LNP, which encodes for multiple antigens, including the spike (S) protein	Ziphius Vaccines and Ghent University

**Table 4:** Recombinant spike protein nanotechnology-based vaccines that are in clinical use in some countries and undergoing preclinical trials in others (adapted from VIPER Group COVID-19 Vaccine Tracker Team [45]).

Vaccine	Company	Phase	Status
Zifivax	Anhui Zhifei Longcom	—	Approved
Noora vaccine	Bagheiat-Allah University of Medical Sciences	—	Approved
Corbevax	Biological E Limited	—	Approved
Abdala	Center for Genetic Engineering and Biotechnology (CIGB)	—	Approved
Soberana 02	Instituto Finlay de Vacunas Cuba	—	Approved
MVC-COV1901	Medigen	—	Approved
Nuvaxovid/NVX-CoV2373	Novavax	—	Approved
CHO cell	National Vaccine and Serum Institute	—	Approved
Razi Cov Pars	Razi Vaccine and Serum Research Institute	—	Approved
SKYCovione	SK Bioscience Co. Ltd.	—	Approved
SpikoGen	Vaxine/CinnaGen Co.	—	Approved
Aurora-CoV	Vector State Research Center of Virology and Biotechnology	—	Approved

## Data Availability

No underlying data was collected or produced in this study.
